# Systematic Review of Important Bacterial Zoonoses in Africa in the Last Decade in Light of the ‘One Health’ Concept

**DOI:** 10.3390/pathogens8020050

**Published:** 2019-04-16

**Authors:** Jonathan Asante, Ayman Noreddin, Mohamed E. El Zowalaty

**Affiliations:** 1 Virology and Microbiology Research Group, School of Health Sciences, College of health Sciences, University of KwaZulu-Natal, Westville Campus, Durban 4000, South Africa; josante33@yahoo.com; 2 Infectious Diseases and Anti-Infective Therapy Research Group, Sharjah Medical Research Institute and College of Pharmacy, University of Sharjah, Sharjah 27272, United Arab Emirates; anoreddin@sharjah.ac.ae

**Keywords:** Zoonosis, livestock, bacteria, antimicrobial resistance, animals, Africa, antibiotics, One-health, epidemiology

## Abstract

Zoonoses present a major public health threat and are estimated to account for a substantial part of the infectious disease burden in low-income countries. The severity of zoonotic diseases is compounded by factors such as poverty, living in close contact with livestock and wildlife, immunosuppression as well as coinfection with other diseases. The interconnections between humans, animals and the environment are essential to understand the spread and subsequent containment of zoonoses. We searched three scientific databases for articles relevant to the epidemiology of bacterial zoonoses/zoonotic bacterial pathogens, including disease prevalence and control measures in humans and multiple animal species, in various African countries within the period from 2008 to 2018. The review identified 1966 articles, of which 58 studies in 29 countries met the quality criteria for data extraction. The prevalence of brucellosis, leptospirosis, Q fever ranged from 0–40%, 1.1–24% and 0.9–28.2%, respectively, depending on geographical location and even higher in suspected outbreak cases. Risk factors for human zoonotic infection included exposure to livestock and animal slaughters. Dietary factors linked with seropositivity were found to include consumption of raw milk and locally fermented milk products. It was found that zoonoses such as leptospirosis, brucellosis, Q fever and rickettsiosis among others are frequently under/misdiagnosed in febrile patients seeking treatment at healthcare centres, leading to overdiagnoses of more familiar febrile conditions such as malaria and typhoid fever. The interactions at the human–animal interface contribute substantially to zoonotic infections. Seroprevalence of the various zoonoses varies by geographic location and species. There is a need to build laboratory capacity and effective surveillance processes for timely and effective detection and control of zoonoses in Africa. A multifaceted ‘One Health’ approach to tackle zoonoses is critical in the fight against zoonotic diseases. The impacts of zoonoses include: (1) Humans are always in contact with animals including livestock and zoonoses are causing serious life-threatening infections in humans. Almost 75% of the recent major global disease outbreaks have a zoonotic origin. (2) Zoonoses are a global health challenge represented either by well-known or newly emerging zoonotic diseases. (3) Zoonoses are caused by all-known cellular (bacteria, fungi and parasites) and noncellular (viruses or prions) pathogens. (4) There are limited data on zoonotic diseases from Africa. The fact that human health and animal health are inextricably linked, global coordinated and well-established interdisciplinary research efforts are essential to successfully fight and reduce the health burden due to zoonoses. This critically requires integrated data from both humans and animals on zoonotic diseases.

## 1. Introduction

Zoonoses are infectious diseases caused by pathogens through the natural transmission between animals and man, directly (through agents such as saliva, blood, mucous and faeces) or indirectly (i.e., through environmental sources and vectors) [1]. Of all known human pathogens, including viruses, bacteria, fungi and parasites, an estimated 61% are regarded as zoonotic, with approximately 73% of emerging and re-emerging infections being considered as zoonoses [2]. Globally, it is estimated that 2.5 billion cases related to zoonotic infections are recorded yearly, resulting in 2.7 million deaths [3]. Zoonotic diseases account for 25% of the infectious disease burden in low-income countries, as poverty increases the risk for zoonotic diseases in communities where people are in close contact with livestock and wildlife [4,5]. The World Health Organization (WHO) estimated that, in 2010, there were 600 million cases of foodborne diseases, 350 million of which were caused by pathogenic bacteria [6]. A combined disease burden is imposed on people in poor areas such as tropical and subtropical Africa, where there is the likelihood of zoonotic diseases coinfection with other pathogenic or infectious diseases, such as malaria, tuberculosis and HIV. These associated factors may increase the severity of diseases and the susceptibility of individuals to infectious zoonotic agents, thus enhancing their spread at the community level [7]. Examples of bacterial zoonoses include anthrax, botulism, plague and tularemia, which are listed in category A warfare agents [8,9]. Bacterial zoonoses listed in category B agents include brucellosis, foodborne agents (*E. coli* O157:H7, salmonellosis and shigellosis), glanders, psittacosis, melioidosis, Q-fever, and typhus fever [9]. Zoonotic pathogens such as *Campylobacter, Salmonella*, *Listeria monocytogenes* and the Enterobacteriaceae family are frequently found in livestock (avian, bovine, caprine, equine, ovine and porcine) as well as in wild animals, pets and rodents, causing foodborne diseases. In immunocompromised populations, such as those with a high prevalence of HIV infection, the occurrence of zoonotic diseases is even higher. HIV infection, by depressing the immune systems leads to increased severity of symptoms of many zoonotic diseases and prolonged illness [1].

The absence of effective human monitoring and surveillance programs for zoonotic diseases coupled with limited laboratory capacities leads to a lack of clinical alertness, resulting in underdiagnoses and the subsequent mismanagement of these diseases. This further presents a challenge in detecting new and re-emerging pathogens early [10,11]. Zoonotic pathogens that tend to cause epidemics are usually given more attention regarding characterisation and policy-making than those that do not, despite the latter group having a major impact on rural communities [8].

The public health burden and socioeconomic effects of zoonotic diseases may vary according to geographical location, with a lack of data on disease burden in developing countries resulting in an underestimation of their impact [8].

Antimicrobial resistance has become a subject of global interest; especially as the use of antimicrobial agents continue to rise in both clinical and veterinary practices [12]. Microorganisms adapt to the effects of antimicrobial agents through numerous mechanisms, to enable them to survive in the presence of therapeutic concentrations of the antimicrobials. Thus, infections caused by pathogenic bacteria have become increasingly difficult to treat, due to the various antibiotic resistance mechanisms deployed by bacteria to evade the effects of antibiotics [12,13].

Humans, animals and the environment are interconnected in a complex and diversified manner. The interaction between humans, animals and the environment means that infections/resistance that originate in humans, animals, foods and farms will predictably lead to the spread of infection/resistant bacteria and/or resistance genes in the environment [13,14]. This dissemination of resistance may be facilitated by excreta coming into contact with soils as well as surface and ground water [14]. Thus, the ‘One Health’ approach seeks to amalgamate human and veterinary medicine, environmental sciences and public health to develop effective surveillance techniques, accompanied by appropriate diagnostic and therapeutic interventions. This holistic and coordinated approach will lead to the enactment of more thorough and effective policies [15].

This is the first timely, comprehensive, and updated systematic review about the significant bacterial zoonotic diseases in Africa over the past decade. The review summarises relevant publications reporting on occurrence, diagnosis and control of bacterial zoonoses in Africa within the last decade. The special focus of this study on Africa is explained by the limited data on disease burden of bacterial zoonoses within the continent, as well as the lack of effective monitoring and surveillance policies/techniques. The majority of African countries are classified as low- and middle-income nations; hence, the risk of disease transmission in communities in close contact with livestock is compounded by poverty. Furthermore, several countries in Africa specifically western and eastern Africa are at high risks of zoonotic diseases, where there are areas characterized by interplay of intense livestock animals, agricultural activities, and poor health services [16]. Furthermore, the risk of disease transmission in communities in close contact with livestock is compounded by poverty. Thus, the review provides important information to fill in the information gap.

## 2. Methods

### 2.1. Systematic Review Protocol

**Figure 1:**
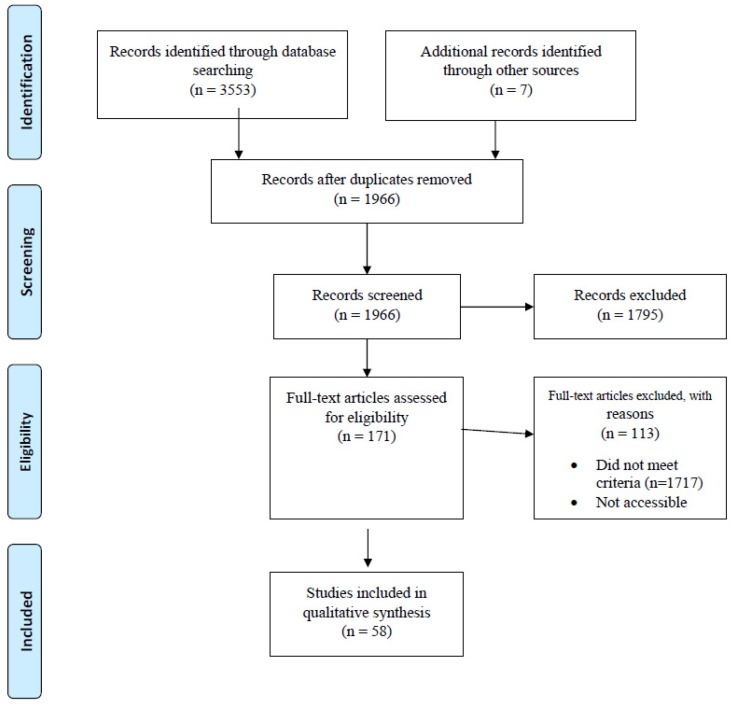
Preferred Reporting Items for Systematic Reviews and Meta-Analyses (PRISMA) flowchart showing search strategy and selection process for the research articles published between 2008 and 2018 used in the current study. Based on the search strategy, 3553 English articles were identified in total. Duplicates were removed.

The systematic review followed the standard systematic review procedures established by the Preferred Reporting Items for Systematic Reviews and Meta-Analyses (PRISMA). The review used the following guidelines: (a) a database search to identify potentially relevant articles, (b) evaluating the relevance of articles, (c) quality assessment and (d) extraction of data, and are summarised in Figure 1.

### 2.2. Search Strategy and Data Collection/Extraction

In August 2018, we searched the English literature published between 2008 and 2018 on three scientific database search engines (PubMed, Web of science and Science Direct) for relevant articles using the search terms (Bacterial zoonoses OR zoonotic bacterial pathogens) AND (Africa) for articles published between January 2008 and August 2018. Other related articles that arose during the search, including bibliographies from selected papers were reviewed and added as additional information sources. Duplicate entries were identified and removed before the final selection of articles. Studies that did not meet the predetermined inclusion criteria were removed and included those outside the scope of Africa, nonbacterial zoonoses, conducted/published before 2008, non-English language, reviews, abstracts and conference proceedings. Citations were compiled and deduplicated using EndNote (Thomson Reuters, New York, NY, USA).

### 2.3. Data Screening

The full texts of retrieved articles were screened for inclusion. Studies were selected for evaluation if they met the following inclusion criteria.

- Any research article published between January 2008 and August 2018 that discusses bacterial zoonoses in Africa in both humans and animals.
- Any article that describes information relating to the occurrence (including outbreaks), diagnosis and control of bacterial zoonoses from any country, as defined by the United Nations (UN), within the stated period. Bacterial zoonoses/zoonotic bacterial pathogens were selected for inclusion based on the classification given by the individual studies.

Articles classified as eligible for inclusion were retrieved in full text format and were assessed using the case definitions specified by the respective studies (Table 1). Only accessible articles were screened. Studies were included if they reported on data from any country in Africa within the United Nations (UN) definition of Africa [17]. Only diseases/pathogens that routinely involve animal to human transmission were considered. Pathogens such as *Escherichia coli* and *Staphylococcus aureus*, which may or may not involve animal reservoirs, were excluded.

### 2.4. Data Analysis

The statistical analysis was carried out using SPSS version 25 [18] and R software version 3.5.2. [19].

### 2.5. Quality Assessment and Data Extraction

Two independent researchers conducted full texts analysis of each publication using a data extraction form to extract predetermined qualitative and quantitative data; inconsistencies were decided by consensus. Data that consisted of sample size, infection prevalence, diagnosis/investigations, disease/pathogen, host/vector, country and year of study/publication were extracted from included eligible articles and compiled. The independent researchers examined eligibility of studies according the following criteria: appropriate description of study design which guaranteed the quality of the methodology, description of population and sample size for epidemiological studies and strength of association for studies reporting on risk for human infection. Articles were excluded if there was insufficient information in the methodology to decide if criteria were met. Studies that satisfied requirements for quality assessment were considered of enough quality to provide evidence of bacterial zoonoses in different host populations or probable predisposing risk factors.

### 2.6. Ethical Approval

This article does not contain any experimental studies involving human participants or animals performed by any of the authors. Parts of the manuscript involving data from ongoing research projects where ethical approvals were obtained from the Animal Research Ethics Committee of the University of KwaZulu-Natal (Reference: AREC 071/017 and AERC 014/018). The field sampling protocols, samples collected from animals and the research were conducted in full compliance with Section 20 of the Animal Diseases Act of 1984 (Act No 35 of 1984) and were approved by the South African Department of Agriculture, Forestry and Fisheries DAFF (Section 20 approval reference number 12/11/1/5 granted to Prof Dr. ME El Zowalaty).

## 3. Results

### 3.1. Data Acquisition

The preliminary database search yielded 3553 results. Manual search identified seven additional articles. Deduplication yielded 1966 unique articles. Reports were considered duplicated if they had the same information in the author, year of publication, name of the peer review, volume issue and page number fields. After removal of papers that did not meet the inclusion criteria, 58 papers were left for data extraction and qualitative analysis (Table 2). These included 15 articles reporting on *Brucella* spp. [20,21,22,31,32,33,34,35,36,37,38,39,40,41,42]; nine reporting on *Leptospira* spp. [22,26,27,28,43,44,45,46,47]; 13 reporting on *Coxiella burnetii* [23,24,25,39,40,41,48,49,50,51,52,53,54]; five on *Mycobacterium bovis* [42,55,56,57,58]; eight on *Rickettsia* spp. [25,53,54,59,60,61,62,63]; five reporting on *Anaplasma* spp. [53,63,64,65,66]; two each on *Bartonella* spp. [67,68] and *Borrelia* spp. [69,70]; one each reporting on *Yersinia pestis* [29], *Bacillus anthracis* [71], *Francisella tularensis* [30], *Ehrlichia canis* [53] and *Burkholderia pseudomallei* [40]; and six studies reporting on other zoonotic pathogens including *Salmonella* [72,73,74,75] and *Campylobacter* [76,77] (Table 2). Fourteen studies reported on human zoonoses, 33 were reports on animals, while 11 studies reported on both humans and animals (Table 2).

These articles reported on the occurrence, diagnostic methods of zoonoses in humans, livestock, companion animals and vectors. The studies varied in terms of methodological designs, sampling methods, sample size and diagnostic criteria. Most of the studies were case reports, while three were outbreak reports [29,31,71]. The risk of sampling bias in retrospective seroepidemiological studies may be significant considering that these studies utilised samples collected or submitted to research laboratories and thus did not provide evidence of random sampling.

**Figure 2:**
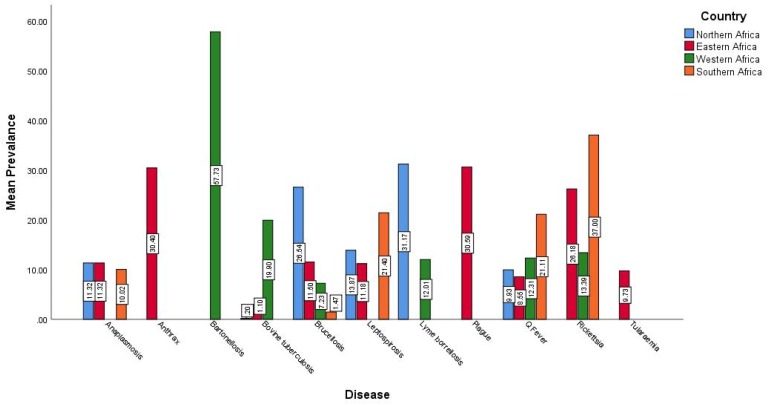
The prevalence of important bacterial zoonotic diseases in different geographic regions in Africa.

The prevalence of different bacterial zoonotic diseases in the four geographic regions in Africa is shown in Figure 2. Bartonellosis was the highest prevalent disease (57.73%) in western Africa and leptospirosis was the highest prevalent (31.17) disease in northern Africa, plague was the highest prevalent (30.59%) in eastern region, while rickettsiosis was the highest prevalent (37%) in southern Africa.

**Figure 3:**
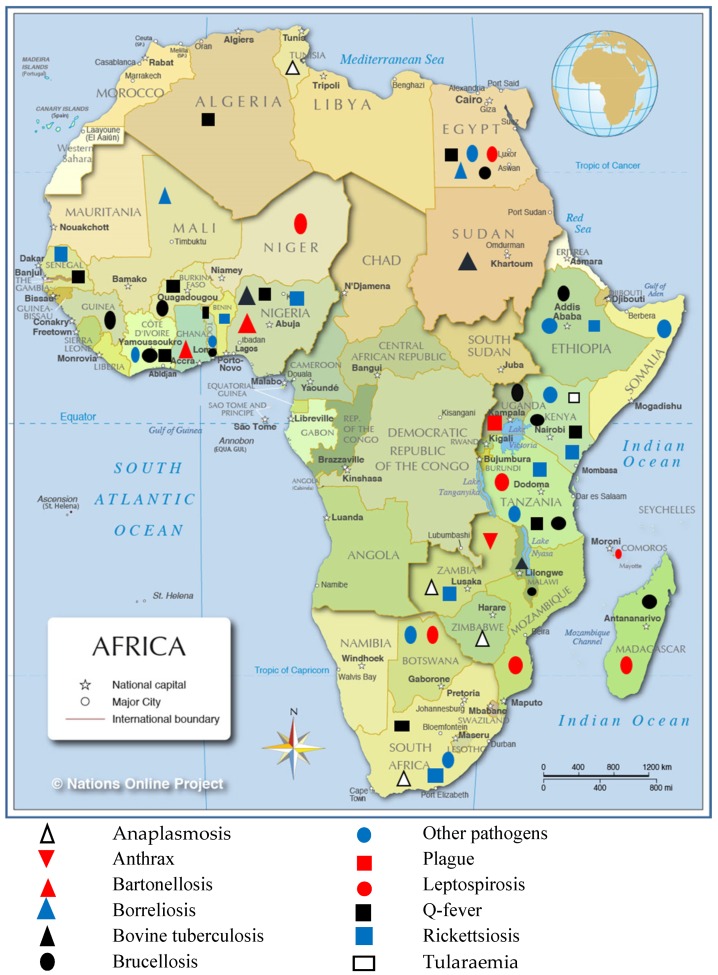
Geographic distribution of important bacterial zoonotic diseases between 2008 to 2018 in Africa. Map of Africa showing locations indicating countries with reported zoonotic diseases and circulation. (Map was reproduced from Nations Online Project.)

As shown in Figure 3, a map of Africa showed the location of the different studies by pathogen in different countries. There was no study that met the inclusion criteria reporting bacterial zoonotic diseases that in central Africa at the time of this review.

### 3.2. Brucellosis

Egypt was the most frequently represented country followed by Kenya and Uganda. The Rose Bengal Test (RBT), complement fixation test (CFT) and enzyme-linked immunosorbent assay (ELISA) were the main diagnostic tests used. Others included culture and biochemical tests, real time PCR (qPCR) and standard microagglutination test (MAT). The prevalence of brucellosis in humans was investigated by four studies including two hospital-based studies [20,21] and two in high risk occupational/population groups [36,39]. Njeru et al. (2016) sought to determine the prevalence of brucellosis in patients in two hospitals in Kenya and to define their clinical characteristics to help clinicians identify cases of brucellosis in regions with limited laboratory capacities. It was reported that 13.7% of samples tested were positive for brucellosis (defined as positive qPCR results or positive RBPT results confirmed by positive ELISA results) [20]. Bouley et al. (2012) also found evidence of brucellosis in 3.5 % of participants screened. There was no diagnosis of brucellosis by the hospital clinical team even though study participants with brucellosis were given antibiotics or antimalarials in the hospital [21]. Using blood samples, Boone et al. (2017), investigated the causes of febrile illness in Madagascar, and found a 1.5% detection rate for *Brucella* [40]. It is the first report of brucellosis in febrile patients reported in Madagascar [40]. Chipwaza et al. (2015) investigated the prevalence of bacterial febrile illnesses in Tanzania, and found that 7.0% and 15.4% showed presumptive acute brucellosis due to *B. abortus* and *B. melitensis*, respectively [22].

In the Uganda study of cattle keepers and consumers of unpasteurised milk, consumption of unpasteurised milk was significantly linked (*p* = 0.004) to seropositivity in one of the districts of the study (Mbarara District). Brucellosis seroprevalence in exposed cattle keepers and consumers of raw milk were 5.8% and 9%, respectively, in this study [36] (Table 2).

Six articles investigated brucellosis in animals including livestock [31,32,33,34,35,37]. In an outbreak investigation in Egypt, one study investigated the molecular profile of *Brucella* isolates and found two different profiles of the *B. abortus* biovar (bv.): one smooth and one rough *B. abortus* strain, with low genetic diversity identified by the molecular typing method and multiple locus of variable number tandem repeats analysis (MLVA-16) [31]. As risk factors for *Brucella* infection, Megersa et al. (2011) found that herd size and age of cattle were found to have played roles in a study investigating the prevalence of cattle brucellosis in traditional animal husbandry practice [32].

Large (odd ratio (OR) = 8.0, 95% CI = 1.9, 33.6) and medium herds (OR = 8.1, 95% CI = 1.9, 34.2) were found to present a higher risk of infection than small herds. One article investigated the prevalence and risk factors for brucellosis in humans and livestock, and found their individual seroprevalence to be 16% and 8%, respectively [38]. Risk factors found to affect the odds for human seropositivity in this study included exposure to goats (adjusted odds ratio (OR) = 3.1, 95% CI = 2.5–3.8), frequent consumption of raw milk (OR = 3.5, 95% CI = 2.8–4.4) and handling of animal hide (OR = 1.8, 95% CI = 1.5–2.2). Again, there was an association between seropositivity in humans and animals, with a six-fold increase observed for humans in households with seropositive animals compared to those without [38].

### 3.3. Q Fever

Three papers investigated the presence of Q fever in human febrile patients [23,24,25]. The study by Angelakis and colleagues (2014) was conducted in five countries—Senegal, Mali, Tunisia, Algeria, Gabon and Morocco—and recorded infection rates of 0.3% and 0.5% in Algeria and Senegal, respectively. For the first time in humans, *Coxiella burnetii* (causative agent of Q fever) genotype 35 was found in a patient in Senegal [24]. In the other study in febrile patients, 16.2% of patients screened had acute Q fever [23] (Table 1). Risk factors for human infection included exposure to goats (OR: 3.74, 95 % CI: 2.52–9.40), cattle (OR: 2.09, 95% CI: 1.73–5.98) and animal slaughters (OR: 1.78, 95% CI: 1.09–2.91). Dietary factors linked with seropositivity were found to include consumption of raw milk (OR: 2.49, 95% CI: 1.48–4.21) and locally fermented milk products (OR: 1.66, 95% CI: 1.19–4.37). Univariate analyses showed no significant association between county of residence, gender, occupations (except herders) and seropositivity. Using ELISA and culture assays, Prabhu et al. (2011) investigated the occurrence of Q Fever in hospitalised febrile patients in northern Tanzania, and found the infection rate to be 5.0% [25].

Five articles probed the presence of Q fever in human and animal hosts [41,48,50,51,52]. Abdel-Moein and Hamza examined vaginal discharges and placental cotyledons from animals that had aborted and found an overall prevalence of 0.9%, with the highest prevalence of Q fever being found in goats (3.4%). A seroprevalence of 19% was detected in the human contacts screened, with a higher prevalence being detected in farmers (30.6%) than veterinarians and veterinary assistants (9.4%) [48]. A higher seroprevalence of 25.71% was found in human contacts in Egypt [50].

In a Gambia study, a 24.9% seropositivity rate in small ruminants and 3.8–9.7% in adults, depending on the ELISA test cut off, was reported [51]. Having at least one seropositive animal in a compound was determined to be a risk factor for human seropositivity (OR: 3.35, 95% CI: 1.09–14.44) [51]. Wardrop et al. (2016) found overall *C. burnetii* seroprevalence in cattle and humans to be 10.5% and 2.5%, respectively [52]. There was no correlation between cattle and human seroprevalence. An article investigated the prevalence of Q fever infection in small ruminants after abortion or the lambing period and found a 14.1% prevalence at individual level and 58.6% at flock level in Algeria [49]. Excretion of bacteria was found in 60% of flocks, with 21.3% of females showing evidence of *C. burnetii* shedding. Dean and colleagues investigated the seroprevalence of Q fever in humans and livestock in Togo, and found that there was a significantly higher *C. burnetii* seroprevalence among the Fulani people, who also had greater livestock contact (45.5%, 95% CI: 37.7–53.6%) [41].

Real-time PCR (qPCR) and ELISA were the most commonly used diagnostic tests. Another test included indirect immunofluorescence assay (IFA) (Table 2).

### 3.4. Leptospirosis

PCR was the most widely used diagnostic method, being used in six out of the eight studies. Other techniques such as culture isolation, MAT and ELISA were also used. Three articles [22,26,27] studied the seroepidemiology of leptospirosis among febrile patients. In a Morocco study, Ribeiro et al. (2017) observed that 1.3% of samples had acute leptospirosis defined therein as a microagglutination test (MAT) > 400, while 10.2% had a presumptive infection, therein defined as IgM-positive/MAT <400. Patients with acute infection had a significantly higher contact with rodents (100%, 5/5) than those with presumptive (39.5%, 15/38) or no infection (41.8%, 138/330) (*p* = 0.031). Although the malaria tests proved negative, 80% of patients with acute leptospirosis were given antimalarial drugs. In addition, 20.9% of the confirmed/presumptive cases of leptospirosis occurred in sub-urban populations. Similarly, Biggs et al. (2011), in their study of leptospirosis in febrile patients in northern Tanzania, observed that 8.8% of paired (acute and convalescent) sera samples were confirmed leptospirosis (defined therein as ≥ four-fold increase in MAT titre) and 3.6% (with ≥1 serum sample available) were classified as having probable leptospirosis (defined therein as MAT titre ≥ 800). The most predominant serotypes were Mini and Australis. There was an association found between Leptospira infection and rural dwelling (OR 3.4, *p* < 0.001) [27]. Chipwaza et al. (2015) found 11.6% seroprevalence of presumptive acute leptospirosis among people presenting with febrile illnesses [22].

In a study in Egypt, Leptospira isolation rates were 1.1%, 6.9% and 11.3% for cows, rats and dogs, respectively, whereas PCR detection rates were 1.1%, 24% and 11.3%, respectively [28]. The human contacts who were tested proved negative by culture isolation and PCR. However, using MAT, the seroprevalence of the human samples was determined to be 49.7%. In that study, six Leptospira serovars (Grippotyphosa Pyrogenes, Icterohaemorrhagiae, Canicola, Celledoni and Pomona) were isolated from cows, rats and dogs. These three species of animals were found in this study to be the most important carriers of leptospirosis in Egypt. Of note is the recovery of some isolates from rats caught from dairy farms and water sources supplying the farms [28]. In a survey of an area with a high reported incidence of human leptospirosis in northern Tanzania, Allan et al. (2018) found no proof of Leptospira in rodents sampled randomly in and around households in the area. However, 7.08% of cattle, 1.20% of goats and 1.12% of sheep from local slaughterhouses carried pathogenic Leptospira infection [47]. Similarly, although *Rattus rattus* and *Mastomys natalensis* are usual rodent reservoirs for Leptospira, Leptopires was not detected in them, although *Leptospira kirschneri* was detected in two rodent species, namely, *Arvicanthis niloticus* and *Cricetomys gambianus*, which are confined to irrigated cultures in the city [46]. The variable number of tandem repeat (VNTR) profiles showed that the leptospires found did not belong to any previously described serovars. The first published report of *L. interrogans* in the Banded mongoose (*Mungos mungo*) and Selous’ mongoose (*Paracynictis selousi*), and the only published report of the pathogen in wildlife in Botswana was reported by Jobbins et al. (2014) [45]. In some cases, the prevalence of Leptospira in animals including bats and other small mammals ranged from 11.7% to 34.6% [43,44].

### 3.5. Bovine Tuberculosis

A bovine tuberculosis infection rate of 0.18% was detected in a Sudan study, with prevalence of 4.5% in slaughtered cattle with caseous lesions [55]. Sa’idu et al. (2014) conducted a study to establish the prevalence of bovine tuberculosis in slaughtered cattle using PCR and Ziehl-Neelsen (ZN) staining and found an overall prevalence rate of 8.3% [56]. In study of bovine tuberculosis in slaughtered cattle in Nigeria, the prevalence of mycobacterium TB was 21.4% (acid-fast bacilli test) and 16.7% (duplex PCR) [58]. The presence of lesions in lungs was highly associated (OR = 52.3; 95% CI: 16.4–191.8) with positive results for acid-fast bacilli (AFB) test compared to those without lesions. A retrospective study at a Nigerian abattoir was conducted with an average yearly bovine tuberculosis prevalence rate of 9.1% detected [57].

### 3.6. Rickettsiosis

Four articles investigated *Rickettsia* spp. in ticks [53,59,60,62] and two in humans [25,61]. Prabhu et al. (2011) investigated the occurrence of spotted fever group (SFGR) and typhus group rickettsioses (TGR) in hospitalised febrile patients in northern Tanzania, and found infection rates to be 5.0%, 8.0% and 0.5%, respectively [25]. Kumsa et al. (2015) investigated the transmission of spotted fever group rickettsiae through ixodid ticks and found an overall prevalence to be 6%. Being the first study to investigate SFG rickettsiae in Benin, Moumouni et al. (2016) found that 29.4% of samples were positive for the SFG rickettsia-specific *ompA* gene, whereas 63.4% were positive by 16S rDNA gene amplification [60]. In Senegal, a study sought to investigate the cause of reported febrile conditions that had tested negative for malaria [61]. The prevalence of spotted fever in all samples was 4.4%, with was no positive sample recorded for typhus group rickettsiae. By sequencing theamplicons, one sample was found to be *R. conorii* [61].

### 3.7. Anaplasmosis

Vlahakis et al. (2018) conducted a study to identify and characterize *Anaplasma* species from dogs in Zambia and found a 9% prevalence of *Anaplasma* spp. as detected by PCR. It is the first study to highlight the prevalence of *Anaplasma* spp. in dogs in Zambia and the first report of *Anaplasma platys* in Zambia [64]. Said et al. (2017) used a restriction enzyme fragment length polymorphism (RFLP) together with a hemi-nested *groEL* PCR method to distinguish between *A. platys* and genetically related strains. Analysis of the sequence variants pointed to infection with an unclassified *Anaplasma platys*-like strains that were genetically related to *A. platys*, with prevalence rates ranging from 3.5% to 22.8% in sheep, goats and cattle [65]. Mtshali and colleagues identified an *Anaplasma phagocytophilum*-like bacterium in 18% of pooled DNA samples [53].

### 3.8. Lyme Borreliosis

Elhelw et al. (2014) investigated the occurrence of borreliosis as an emerging zoonotic disease and its zoonotic potential in Egypt [69] and found *Borrelia burgdorferi* in the animals screened. In addition, the *OspA* gene (outer surface protein A gene) and anti-*B. burgdorferi* IgM were detected by PCR and ELISA respectively in human contacts. The use of culture techniques to isolate *B. burgdorferi* showed low sensitivity as shown by the recovery of only one isolate out of seven samples cultured, while 26.6% of febrile human blood samples tested were positive by PCR, and 15 out of 15 serum samples tested positive for IgM ELISA. The human contacts had been exposed to tick bites, which suggests a possible zoonotic transfer. In Mali, Borrelia seroprevalence of 11.0% and 14.3% in rodents and shrews, respectively, was observed, with 2.2% of animals displaying active spirochete infections at the time of capture [70].

### 3.9. Bartonellosis

In a first report on the occurrence of *Bartonella* spp. in bats and bat flies from Nigeria, 51.4% of bat blood samples and 41.7% of bat flies tested were positive for *Bartonella* spp. DNA [67]. The prevalence by culture of *Bartonella* spp. among five bat species ranged from 0% to 45.5% [67]. Of 137 adult bat flies studied in Ghana, 66.4% were positive for Bartonella DNA [68].

### 3.10. Plague

In a suspected plague outbreak in Uganda, 31% (78 out of 255 suspected cases) of cases were confirmed as plague [29]. The study found a correlation between reports of human plague and a large number of dead rats in a village. Close contacts with rodents, lack of appropriate antibiotics and a delay in seeking medical help contributed to the menace of human plague in the area where the study was conducted [29].

### 3.11. Tularaemia

Among febrile patients seeking treatment at remote hospitals in northeastern Kenya, 9.7% were seropositive for *Francisella tularensis* by ELISA, while 3.7% were confirmed by Western blotting [30]. Most of the febrile cases that tested positive to tularaemia were not recognised by clinicians and the appropriate treatment protocol was not therefore followed. Indeed, most cases were treated with antimalarial agents and/or beta-lactam antibiotics.

### 3.12. Anthrax

In the light of a suspected outbreak of anthrax in Zambia in 2011, a study to investigate the cause was initiated [71]. Human, hippopotamus and soil samples were screened by culture and PCR methods. It was found that 30.4% of samples were culture-positive. All isolates tested were resistant to vancomycin, but showed 100% susceptibility to the penicillins [71].

### 3.13. Others

#### 3.13.1. *Salmonella*

In a study probing antimicrobial resistance profile and serotypes of porcine *Salmonella* isolates from Kenyan slaughterhouses, 13.8% were *Salmonella* positive, while 7.1% of isolates tested showed multidrug resistance [72]. Resistance to tetracycline, ampicillin, chloramphenicol and streptomycin were found to be mediated by the *tet*(A), *bla*_TEM_, *catA1* and *strA* genes, respectively [72]. An Ethiopia study recorded a high multidrug resistance value of 36.7% (to seven or more drugs tested) in *Salmonella* isolated from dairy cattle [73]. In a study to determine the prevalence, antimicrobial susceptibility profiles and serotype distribution of faecal *Salmonella* from apparently healthy dogs, Kiflu et al. (2017) found a *Salmonella* carriage rate of 11.7% in dogs screened [74]. Fourteen *Salmonella* serotypes were detected, with the most dominant ones being *S. bronx* (16.7%), and *S. newport* (14.3%) and 9.5% for each of *S. typhimurium*, *S. indiana*, *S. kentucky*, *S. saintpaul* and *S. virchow*. There was an association between *Salmonella* infection and diarrhoeal symptoms in the past 60 days. Highest antibiotic resistance rates were shown against oxytetracycline (59.5%), neomycin (50%) and streptomycin (38.1%), with 45.2% of isolates showing resistance to three or more of the 16 antibiotics tested [74]. Ahmed et al. (2016) detected the virulence genes *stn, avr*A, *mgt*C, *inv*A and *bcf*C in all screened isolates of *Salmonella enterica* serovar Typhimurium [75]. Antibiotic resistance frequencies detected were as follows; gentamicin (30%), ampicillin and tetracycline (53.3%, each), streptomycin (56.7%) and trimethoprim–sulfamethoxazole and chloramphenicol (73.3%, each). Frequencies of resistance genes discovered in *Salmonella typhimurium*; *sul*1 (96.7%), *tet*A(A) (60%), *tet*A(B) (20%), *flo*R (73.3%), *aad*A1 (46.7%), *aad*A2 (63.3%), *bla*TEM (53.3%), *aad*B (6.7%) and *aad*C (23.3%) [75].

#### 3.13.2. *Campylobacter*

A study was conducted to determine the antimicrobial resistance profile and epidemiology of *Campylobacter* isolated from humans in Tanzania [76]. The prevalence of *Campylobacter* infection in human samples was 11.4%. A high resistance rate was found against erythromycin (84.3%) and azithromycin (89.6%) whereas a relatively low resistance rate of 22.1% was found against ciprofloxacin [76]. In a Botswana study, phylogenetic analysis showed that *Campylobacter* spp. from different poultry and human sources were highly related [77].

## 4. Discussion

In Africa, zoonotic diseases remain to be largely neglected by public health and veterinary services, despite causing a substantial health burden in several countries. This work intends to systematically review data on the most important bacterial zoonoses in Africa, within the period of 2008 to 2018, focusing on the presence, prevalence estimates, causative pathogens, control strategies and risk for human infection. We found 58 studies/reports on 29 countries, which were considered of adequate quality to provide estimates of burden of disease or pathogen, with Egypt (eight), Kenya (seven) and Tanzania (six) being the most represented. We found no reports on zoonotic diseases from central African countries eligible to the inclusion criteria. The distribution of bacterial zoonoses studies in the current study was shown in Figure 3 and was found to be in line with previously reported burden of zoonotic diseases in Africa [78]. Although several bacterial zoonoses such as brucellosis, foodborne diseases, Q-fever, and tuberculosis were reported from countries in central Africa [78,79], we found no reports that were eligible to the inclusion criteria on bacterial zoonotic diseases in this region. The current study reviewed data on the evidence of various zoonoses in humans, multiple species of animals, vectors and the environment. Fourteen reports studied possible bacterial zoonoses in humans (including patients visiting hospitals and high-risk groups), 33 reports investigated zoonoses in animals, whereas 11 reports investigated zoonoses in both humans and animals. Nine reports observed the possible roles of vectors in the transmission of bacterial zoonoses. Vector-borne zoonotic bacterial pathogens carried by vectors (ticks, fleas and bat flies) in this study include *Borrelia* spp., *Rickettsia* spp., *C. burnetii*, *Anaplasma* spp. and *Bartonella* spp. The lack of disease surveillance studies and control programs at the national level in most countries introduces a knowledge gap, and makes it difficult to estimate representative disease burden and thoroughly investigate pathogen transmission dynamics. Thus, more national level epidemiological studies ought to be undertaken to bridge this knowledge gap. The epidemiological picture of zoonotic diseases on the African continent is evolving. The prevalence of zoonotic diseases/pathogens summarised in this review must be interpreted with caution, as many of the studies were conducted within specific geographical and occupational settings/groups and may not be extrapolated to the general population. The changing scenes of rapid urbanisations in various countries may translate to the changing epidemiology of zoonotic diseases.

Considering the complex interrelatedness between humans, animals and the environment, any intervention that seeks to tackle the problem of bacterial diseases and antimicrobial resistance from a non-holistic, single focus point of view is bound to fail. The ‘One Health’ approach seeks to amalgamate and improve the efforts of clinicians, veterinarians, environmentalists, agricultural and public health officials to develop effective surveillance techniques, accompanied by appropriate diagnostic and therapeutic interventions. This holistic and coordinated approach will lead to the enactment of more thorough and effective policies. The achievement of true One-health approach depends of the recognition of the complex interplay between human health, domestic, wild animals, and the environment [78,80,81,82]. It is crucial to implement the one-health components in low-income and resource-limited countries in Africa to tackle and reduce the increasing threats of bacterial zoonotic infectious diseases [16,83,84,85]

### 4.1. Brucellosis

Diagnostic methods most commonly used for brucellosis in developing countries are serologic assays based on rapid slide agglutination tests, albeit the poor specificity of these tests limits their usefulness. Other diagnostic techniques such as ELISA and PCR, were used by most studies on brucellosis [20,36,38,86], are more specific and sensitive, proffering a better correlation with clinical observations, although the latter may not be readily available in many developing countries [87]. The precision of serodiagnosis depends on the presence of antibodies in the serum, and infected animals with low concentrations of antibodies, or no antibodies at all in serum, are therefore likely to present as negative even though they may be infectious [31]. In addition, PCR has the benefit of facilitating the differentiation of *Brucella* genotypes. Considering that the diagnosis and clinical management of febrile illnesses in most developing countries are done empirically, resulting in inaccurate treatment, it is essential to augment the capacity of laboratories to improve the diagnosis accuracy and treatment reliability. This point is highlighted by the fact that 43.2%, 20.5% and 8.2% of patients with brucellosis in the study by Njeru et al. (2016) were diagnosed with typhoid fever, malaria and pneumonia, respectively [20]. In Tanzania, as is the case in many developing countries, brucellosis is an underdiagnosed/misdiagnosed and undertreated disease with no standard treatment protocol usually followed in hospitals, as evinced by the misdiagnosis of it as malaria and pneumonia [21]. The absence of specific symptoms makes it difficult to distinguish brucellosis from several other febrile illnesses occurring in the same geographical area. There is the need for heightened clinical alertness and laboratory capacity building to ensure prompt and accurate diagnoses to aid in the detection and subsequent management of brucellosis in this part of the world. Nasinyama et al. (2014) observed that cELISA test had a sensitivity and specificity of 98.3% and 99.7%, respectively, and is valuable for observing the effectiveness of treatment, prognosis and clinical conditions [36]. Although no single diagnostic test is ideal, with reference to specificity and sensitivity, the standard tube agglutination test (STAT) was preferred in such environments. The limitation of STAT is the long turnaround times, making it unsuitable for seroepidemiological studies, where multiple samples need to be investigated, or in hospital laboratories, where brucellosis therapy has to be initiated quickly. Thus, less time-consuming and faster turnaround diagnostic methods, such as Competitive Enzyme-Linked Assay (cELISA), may need to be used [36].

Although brucellosis has been well recorded in nomadic herdsmen in rural sub-Saharan Africa, owing to their being in close contact with infected animals [88], Bouley et al. (2012) found no association of note between brucellosis and rural residence. While brucellosis prevalence is generally higher in northern Africa [89,90], its seroprevalence ranges from 3 to 8% in sub-Saharan Africa [91]. Despite the implementation of control regimes and strategies, brucellosis remains pervasive in Egypt. Despite immunisations with *Brucella* (*B*.) *abortus* RB51 vaccine, a rise in abortions suspiciously caused by *Brucella *was observed in a dairy cattle herd. The disease has serious economic implications resulting from abortions, infertility and decreased milk production, thus necessitating the implementation of surveillance and control strategies to forestall the socioeconomic effects in both developed and developing countries where the disease is endemic. The prevention, control and eradication strategies against brucellosis usually involve vaccination programmes which employ live, attenuated vaccines as they can elicit long-term cell-mediated immunity [92]. Serological testing and the subsequent culling of seropositive animals are crucial interventions in the adequate control of zoonoses in developing countries.

A large herd size leads to increase in stocking volume, thus exposing more animals to infection [93], as demonstrated by Megersa et al. (2011) [32]. *Brucella* infection in livestock husbandry practice poses zoonotic threats to the public due to close contact with animals, assisting in parturition and the consumption of unpasteurised milk. The study by Osoro et al. (2015) highlights a ‘One Health’ approach to tackling the menace of brucellosis by concurrently looking into the prevalence of brucellosis in both humans and their livestock in the same household [38]. This approach allows for identification and assessment of risk factors for transmission and gives a more complete epidemiological picture and delineates the factors at play at the human–animal interface [38].

### 4.2. Q Fever

Q fever is a common cause of febrile illness in Kenya, but it is underestimated [23]. There is a low level of clinical suspicion, with most febrile patients admitted to hospitals given standard empirical treatments that typically include antimalarials and penicillin antibiotics. Even though Njeru et al. (2016) reported a high Q fever prevalence rate of 16.2%, the most common working diagnosis by clinicians documented in this group was typhoid fever (45.1%), followed by acute respiratory infections/pneumonia (37.6%), malaria (6.9%) and fever of unknown origin (10.4%) [23]. There are indications of increasing cases of severe febrile illnesses of under-recognised zoonotic sources facing clinicians, but diagnostic tools for such conditions are lacking in many African countries [94], leading to overdiagnosis of familiar febrile illnesses even when there is no diagnostic evidence to back.

Bok et al. (2017) determined that having at least one seropositive animal (small ruminant) in one’s compound was a risk factor for human seropositivity [51], highlighting the relationship between seropositivity and closeness of contact with infected animals. Other studies found risk factors for human infection included exposure to goats, cattle and animal slaughters. Dietary factors linked with seropositivity were found to include consumption of raw milk and locally fermented milk products [23].

The use of point-of-care testing in health care centres will inform treatment and decrease the possibility of wrongful diagnosis and inappropriate treatment in febrile patients seeking treatment at health centres. As shown by Angelakis and colleagues, real-time PCR, which is less time-consuming than conventional PCR, can come in handy in decreasing delays in diagnosis, thereby facilitating prompt treatment [24]. Even though the immunofluorescent assay test (IFAT) is considered the gold standard for serological detection of Q fever, it still falls short and requires highly experienced technicians [52,95]. There is the likelihood that some infected animals may shed bacteria without having antibodies thus they may be classified as negative by serology, leading to an underestimation of associated risk factors. Analysing animals for the shedding ability would partly provide a solution. Excretion of bacteria was found in 60% of flocks by one study [49], presenting a significant risk in the spread of the disease especially to humans.

### 4.3. Leptospirosis

The possible role of rodents in the transmission of the disease was underscored by the observation that patients with acute infection had a significantly higher contact with rodents than those with presumptive or no infection [26]. Also, a study found an association found between Leptospira infection and rural dwelling (OR 3.4, *p* < 0.001) [27]. Again, a worrying case of misdiagnosis and subsequent inappropriate treatment was observed, as 80% of patients with acute leptospirosis were given antimalarial drugs by prescribers in Mozambique [26].

There may be a gradual expansion in the occurrence of leptospirosis from the typical rural communities to sub-urban communities as evidenced by the fact that 20.9% of the confirmed/presumptive cases of leptospirosis occurred in sub-urban populations in the Mozambique study [26]. This shift has been demonstrated to be associated with inadequate sanitation, poor hygiene, rise in rodent population and poor disposal of solid waste. With the rising trend of rural–urban/sub-urban migration, coupled with attendant problems such as frequent floods and global warming, it can be predicted that leptospirosis will pose a great public health threat in the near future. This prediction is particularly relevant for Mozambique as the country has been rated as the third most vulnerable country to extreme climate events in Africa [26].

MAT as a technique may help provide hints of animal reservoirs by showing the common serogroups prevalent in a specific locality, although the technique is not serovar-specific [96]. In a study by Samir et al. (2015), there was a disagreement between PCR and MAT results in evaluating seroprevalence in humans. This highlights the need for increased surveillance and well-planned prevention and control programs, particularly those that target animals as the source of infection to eradicate the disease. Vaccination programs targeted at livestock and pets would help reduce the disease burden in animals, and reduce environmental contamination and exposure of humans to the pathogen. The detection of *Leptospira interrogans* in banded mongoose (*Mungos mungo*) in Botswana is an important finding, as they are also found frequently in central and eastern Africa, and are thus important to public health [45]. Situations that force humans, domestic animals and wildlife animals to share sources of water put populations at risk of outbreaks, while flooding rivers may carry soil contaminated with urine [45].

### 4.4. Bovine Tuberculosis

In cattle, post-mortem and bacteriological examinations of suspected lesions are important ways of confirming the presence of bovine tuberculosis. The mycobacterial species concerned are characterised by molecular methods, while the specificity of diagnosis may be improved by histopathological examination. As accurate diagnosis is key, routine culturing and other reliable diagnostic techniques are required to make definitive diagnosis, to help fashion control programs [55]. Phenotype-based characterisation of mycobacteria is laborious and less reproducible compared to molecular detection techniques, such as PCR, which has a higher sensitivity and specificity, and is faster and more reliable [55]. However, conventional detection methods remain useful in many developing countries, as molecular techniques may not be readily available due to cost. It was found that PCR showed high sensitivity and specificity, and thus can be relied upon to confirm the results of tests from Ziehl-Neelsen (ZN) staining, tuberculin skin test and postmortem, particularly as these tests are liable to give false positives [56].

### 4.5. Rickettsiosis

Ticks and mosquitoes are known to be the two main vectors of several human and animal pathogens [97], with recent studies indicating an increase in the number of tick-borne pathogens of humans and animals [59]. The occurrence of spotted fever group (SFG) rickettsiae differs according to the location and tick gender. The pathogen *Rickettsia felis*, commonly borne by fleas, causes flea-borne spotted fever, which can manifest as a mild to moderate disease, symptoms of which include cutaneous rash, fever, neurologic and digestive signs. Socolovschi et al. (2010) investigated the cause of reported febrile conditions that had tested negative for malaria [61]. The prevalence of spotted fever in all samples was 4.4%, with *R. felis* infection possibly being responsible for many cases of uneruptive fevers of unknown origins particularly those accompanied with digestive, neurologic and respiratory signs [61]. Vector-borne bacterial zoonoses have complex epidemiology and ecology, meaning factors such as weather and climate can affect transmission cycles, making them hard to control [98].

### 4.6. Anaplasmosis

Ruminants and rodent species are known natural hosts for *Anaplasma phagocytophilum*, with humans and dogs being considered accidental hosts. However, *A. platys* naturally infects dogs, and is thought to be transmitted by the *Rhipicephalus sanguineus* group of ticks [99]. The close bond shared between humans and dogs can facilitate the transmission of pathogens between them, as dogs spend time outdoors and also closely associated with humans, which means that they are a good source of tick-borne infections [64]. The first study to highlight the prevalence of *Anaplasma* spp. in dogs in Zambia [64] is important from the viewpoint of ‘One Health’, as it recognises dogs as important reservoirs of zoonotic pathogens, thus increasing the risk for human infection. Increased sensitisation among veterinarians and dog owners is essential. Other measures such as use of insect repellents, insecticide treatment of pets and frequent tick checks on pets after outdoor activity in high risk communities will help check the spread of vector-borne pathogens [98].

### 4.7. Lyme Borreliosis

Lyme borreliosis is mainly transmitted through Ixodes ticks to mammalian hosts. The main reservoirs for the disease are deer and small rodents especially mice. Elhelw et al. (2014) in their study of Lyme borreliosis in Egypt found the *OspA* gene (outer surface protein A gene) and anti-*B. burgdorferi* IgM by PCR and ELISA, respectively, whereas culture identification techniques showed a low sensitivity for the recovery of *Borrelia burgdorferi* isolates in humans [69]. Thus, it would be more tenable to rely on PCR and ELISA when dealing with this pathogen. The prior exposure of human contacts to tick bite in that study, suggests a possible zoonotic transfer.

### 4.8. Bartonellosis, Plague, Tularaemia and Anthrax

Bartonella species are mostly thought to be transmitted by arthropod vectors. The detection of bacterial DNA, however, does not necessarily indicate that the organism is viable or that the vector is capable of transmitting the pathogen [68].

Plague occurs worldwide, although most suspected human cases are reported in developing countries, with sub-Saharan Africa accounting for more than 95% of the human cases worldwide [29]. In light of the fact that rodents and fleas are natural reservoirs of *Yersinia pestis*—the causative pathogen for plague [29]—Forrester and colleagues found a correlation between reports of human plague and a large number of dead rats in a village, which is unsurprising considering that close contact with infected rodents is a risk factor for the disease. Even though plague is a less frequent zoonosis, it still retains public health significance because of its epidemic potential [98].

As was observed in other studies, most febrile cases that tested positive to tularaemia in a Kenya study [30] were not recognised by clinicians and hence the appropriate treatment protocol was not followed. Indeed, most cases were treated with antimalarials and/or beta-lactam antibiotics which are ineffective against the pathogen of concern.

In developing countries, where there is high level of interaction at the human–animal interface, anthrax, caused by *Bacillus anthracis*, continues to pose public health threats [71]. Testing the susceptibility of bacterial isolates to some antibiotics, Hang’ombe et al. (2012) in an investigation of a suspected anthrax outbreak, observed that all tested isolates were sensitive to the antibiotics used (including ciprofloxacin and doxycycline), except vancomycin. Ciprofloxacin and doxycycline are recommended by the US Centers for Disease Control and Prevention (CDC) as first line treatment for anthrax [100].

### 4.9. Other Zoonotic Pathogens

Other bacterial zoonotic pathogens, including *Salmonella* spp. and *Campylobacter* spp., which can be transmitted between livestock and humans, were reported by various studies.

Salmonellosis is one of the most common foodborne zoonoses in developing and industrialised countries [72]. The presence of *Salmonella* in food animals and animal products presents a food safety threat [72]. Food safety measures need to be intensified particularly as multidrug resistant pathogenic strains are increasing.

*Campylobacter* frequently colonizes different species of animals asymptomatically, but produces acute and self-limiting intestinal infections in humans [76], with undercooked and raw poultry meat having been particularly found to be culpable. In a study by Komba et al. (2015), *Campylobacter* isolates showed 84.3% resistance to erythromycin, which is worrying, considering that erythromycin together with ciprofloxacin are the antibiotics of choice in the treatment of severe, nonself-limiting *Campylobacter* infections such as septic arthritis, bacteremia and prolonged enteritis [76,101]. Salmonellosis and campylobacteriosis are reported as the commonest foodborne bacterial zoonoses in Europe with eggs and mixed foods as the most culpable food sources [98]. However, prevalence data for these two zoonoses are lacking in Africa. The overuse of antibiotics (mainly as growth promoters) in animal husbandry, coupled with the close contact of humans and farm animals, facilitates the emergence of resistant zoonotic bacterial pathogens. Indeed, studies elsewhere have shown that resistance in pathogenic zoonotic bacteria and/or changes in faecal microbiota increases shortly after the introduction of antibiotics in veterinary practice [102,103,104]. Stricter controls concerning the nontherapeutic use of antibiotics in animal husbandry are required.

### 4.10. Limitations of the Data

The lack of surveys on zoonoses at the national levels, as well as individual studies not being representative enough, might affect the true estimates of zoonoses in individual countries and across the continent. Furthermore, individual reports included in this study have not factored in confounding bias, which may affect the true estimates.

## 5. Conclusions

Bacterial zoonotic diseases pose a significant burden in Africa, although the actual socioeconomic burden is unknown. Interactions at the human–livestock and human–wildlife interfaces contribute to the transmission of zoonoses, with a wide range of hosts and vectors playing roles. Bacterial zoonoses have a dual impact on both livestock production systems and human health. The lack of diagnostic tests and clinical awareness for many zoonotic diseases in most parts of Africa is worrying, being reflected in the low levels of diagnoses on the continent in clinical settings. A ‘One Health’ approach, which involves the concerted efforts of veterinarians, physicians, public health workers and epidemiologists, is essential in the policy schemes that are aimed at controlling and preventing the transmission of such diseases.

## Figures and Tables

**Table 1:** Case definitions in humans and animals.

Disease	Criteria			Reference
**Brucellosis**	**Confirmed**	**Probable**		
	Positive qPCR results or positive RBPT results confirmed by positive ELISA results			[20]
	Blood culture or a ≥4-fold increase in microagglutination test titre	Single reciprocal titre ≥160		[21]
	**Presumptive acute brucellosis**	**Probable prior brucellosis exposure**		
	Positive ELISA IgM antibodies result for *B. abortus*	Positive anti-*Brucella* IgG ELISA result		[22]
**Q fever**	**Acute Q fever**	**Chronic Q fever**	**Exposed**	
	Evidence criteria consistent with clinical evidence and supported by laboratory results indicated by elevated levels of ELISA IgG phase I and phase II antibodies and confirmed by IFA assay showing *C. burnetii* phase II antibodies titres of >1:128 or qPCR detection of *Coxiella* DNA	Cases with elevated ELISA IgG phase I antibodies and IFA assay phase I antibodies titres of ≥1:800.		[23]
	Clinical symptoms confirmed by qPCR targeting the IS1111 andIS30A spacers			[24]
	A ≥4-fold increase in immunoglobulin (Ig) G IFA titre to *Coxiella burnetii* phase II antigen		Titre ≥1000 to Phase I antigen or ≥64 to Phase II antigen on either sample defined Q fever exposure among those serum samples not meeting the case definition for acute Q fever	[25]
**Spotted fever group rickettsiosis (SFGR) and typhus group rickettsiosis (TGR)**	**Acute**		**Exposed**	
	A ≥4-fold increase in IgG IFA titre to *Rickettsia conorii* or *Rickettsia typhi* antigen		Titre to *R. conorii* or *R. typhi* ≥64 defined SFGR or TGR exposure, respectively, among samples that did not meet case definition for acute	[25]
**Leptospirosis**	**Acute**	**Presumptive acute leptospirosis**	**Probable prior leptospirosis exposure**	
	A MAT cut-off titre of ≥1:160	Positive IgM antibodies result *for Leptospira*	Positive anti-Leptospira IgG ELISA result	[22]
	Microagglutination test (MAT) > 400	IgM-positive/MAT < 400		[26]
	**Confirmed *Leptospira* infection**	**Probable leptospirosis**	**Exposure to pathogenic leptospires**	
	A ≥ four-fold increase in MAT titre	MAT titre ≥ 800	Titre ≥ 100	[27]
	Positive culture detection of Leptospira and/or positive PCR-specific assay for pathogenic *Leptospira* spp. Also, pathogenic serovar titre ≥ 200 considered positive by MAT			[28]
**Plague**	**Confirmed**	**Suspected**	**Probable**	
	clinically compatible acute illness with isolation of *Y. pestis* from a clinical specimen OR > 1 positive antibody titre against the F1 antigen of *Y. pestis*	Clinically compatible acute illness without laboratory confirmation	Suspected case linked epidemiologically to a confirmed case OR suspected case with further nonconfirmatory laboratory evidence of plague infection	[29]
**Tularaemia**	**Positive**	**Negative**		
	optical density > 0.25 (ELISA)	optical density <0.20 were considered negative		[30]

**Table 2:** Diagnoses, sources and study outcomes of bacterial zoonoses in Africa between 2008 and 2018.

Country	Period of Study	Year of Publication	Disease/Pathogen	Host/Vector/Source	Diagnostic Test/Investigations	Number of Animals/Humans/Samples Tested	Study Outcome/Disease Frequency/Seroprevalence	Reference
**NORTHERN AFRICA**								
Algeria	2011–2013	2016	Q fever (*Coxiella burnetii*)	Small ruminant flocks (aborted females)	Indirect ELISA, real time PCR (q-PCR)	494 samples (227 sera and 267 genital swabs)	*C. burnetii* seroprevalence was 14.1%. Bacterial excretion observed in 60% of flocks whiles 21.3% of females showed evidence of *C. burnetii* shedding.	[49]
Egypt	2008–2009	2014	Lyme borreliosis/*Borrelia burgdorferi*	Cattle, dogs, humans	Culture, PCR, enzyme-linked immunosorbent assay (ELISA)	92 samples (15 human blood samples, 25 cattle, 26 dog blood samples and 26 ticks)	24 out 77 non-human samples (51 blood and 26 tick) positive for the *OspA* gene. All human serum samples positive for IgM against *B. burgdorferi*	[69]
Egypt	2014	2014	*Brucella* spp.	Cattle, buffaloes	iELISA, qPCR	215 unpasteurised milk samples	34 (16%) samples were positive for anti-*Brucella* antibodies (iELISA) whiles qPCR detected *Brucella*-specific DNA from 17 (7.9%) milk samples.	[33]
Egypt	2015	2015	*Brucella abortus*	Cows, buffaloes, Egyptian Baladi goats and ewe	RBT, CFT, ELISA	25 serum samples from aborted animals	All 25 samples positive by PCR, but 10 positive by serology. *B. abortus* DNA was detected in all serum samples taken from buffaloes, goats, ewe and cows.	[35]
Egypt	2015	2015	Leptospirosis	270 rats, 168 dogs, 625 cows, 26 buffaloes, 99 sheep, 14 horses, 26 donkeys and 22 camels, humans and water sources	Culture, PCR and MAT.	Samples from 1250 animals, 175 human contacts and 45 water sources	Leptospira isolation rates were 6.9%, 11.3% and 1.1% for rats, dogs and cows, respectively. PCR detection rates were 24%, 11.3% and 1.1% for rats, dogs and cows, respectively.	[28]
Egypt	2016	2016	Bovine brucellosis (*Brucella abortus*)	cattle	Culture and biochemical tests, PCR, RBT, serum agglutination test (SAT), complement fixation test (CFT)	Samples selected from an outbreak in which 21 out of 197 pregnant, previously vaccinated cows aborted.	Two *B. abortus* biovar (bv.) 1 smooth and two *B. abortus* rough strains detected	[31]
Egypt	2015	2016	*Salmonella enterica* serovar Typhimurium	Chicken meat and humans	Culture, antimicrobial sensitivity testing, PCR.	700 samples (500 fresh chicken meat samples, 100 hand swab and stool samples each from workers)	Seventy-eight (11.1) of samples were *Salmonella* isolates, of which 18 were from humans and 60 from chicken samples). The virulence genes *stn*, *avr*A, mgtC, *inv*A and *bcf*C were detected in all screened isolates	[75]
Egypt	2017	2017	Q fever (*Coxiella burnetii*)	Small ruminants and humans	Serological assay	183 samples (109 sheep, 39 goats and 35 humans)	Seroprevalence of *C. burnetii* IgG antibodies were 25.71%, 28.20% and 25.68% in humans, goats and sheep, respectively	[50]
Egypt	2016	2017	Q fever (*Coxiella burnetii*)	27 sheep, 29 goats, 26 cattle, 26 buffaloes	Nested PCR, ELISA	108 aborted dairy animals, 56 human contacts	3.4% prevalence in goats, 0.9% overall prevalence, 19% prevalence in humans examined	[48]
Sudan	2007–2009	2013	Bovine tuberculosis	Cattle	Microscopy, culture, PCR	6680 bovine carcasses	Bovine TB infection rate was 0.18%.	[55]
Tunisia	2015	2017	*Anaplasma platys*-like infection	Goats, sheep and cattle	Restriction Enzyme Fragment Length Polymorphism (RFLP) assay, hemi-nested *groEL* PCR	963 domesticated ruminants	Prevalence rates were 22.8, 11 and 3.5% in goats, sheep, and cattle, respectively.	[65]
**WESTERN AFRICA**								
Benin	2011	2016	Spotted fever group rickettsiae	*Amblyomma variegatum*	PCR.	910 ticks	Nearly one-third (29.4%) of samples (267/910) were positive for the SFG rickettsia-specific *ompA* gene, whereas 63.4% were positive by 16S rDNA gene amplification	[60]
Burkina Faso, Togo	2011–2012	2013	Brucellosis and Q Fever	Humans and livestock	RBT, ELISA, immunofluorescence assay (IFA)	683 people, 596 cattle, 465 sheep and 221 goats, 464 transhumant cattle from Burkina Faso	7 *Brucella* seropositive in humans, 9.2% seropositivity in village cattle, 7.3% in transhumant cattle and 0% in small ruminants	[41]
Côte d’Ivoire	2012-2014	2017	Brucellosis, Q Fever	Livestock and humans	Rose Bengal Test (RBT), indirect and competitive ELISAs for the respective pathogens	633 cattle, 622 small ruminants and 88 humans	Human seroprevalence for *Brucella* spp. was 5.3%., 4.6% seroprevalence in cattle adjusted for clustering. Q Fever seroprevalence was 13.9% in cattle, 9.4% in sheep and 12.4% in goats.	[39]
The Gambia	2014	2017	Q fever	Humans and small ruminants	ELISA, PCR	599 human serum and 615 small ruminant serum samples	24.9 seropositivity rate in small ruminants, and 3.8–9.7% in adults depending on ELISA test cut off	[51]
Ghana	2012	2012	*Bartonella* species	Bat flies	Culture and PCR analysis	137 adult flies	*Bartonella* DNA was found in 66.4% of specimen	[68]
Guinea	2011	2014	Brucellosis	Cattle	RBT, CFT	300 serum samples	29/300 RBT-positive, 26 of which were confirmed by CFT. Mean brucellosis prevalence for 2 communities was 8.67%.	[37]
Mali	2007–2011	2012	Tick-borne relapsing fever/ *Borrelia crocidurae*	*Ornithodoros sonrai* ticks, rodents and shrews.	Microscopy, serology (immunoblot)	663 rodents, 63 shrews and 278 ticks	Seroprevalence of *Borrelia* was 11.0% and 14.3% in rodents and shrews respectively	[70]
Niger	2009–2011	2015	Leptospirosis	*Arvicanthis niloticus*, *Cricetomys gambianus*, *Mastomys natalensis*, *Mus musculus* and *Rattus rattus*	qPCR, 16S-based metabarcoding, rrs gene sequencing, VNTR	578 samples	Leptospires not detected in *R. rattus* and *Mastomys natalensis*, but *Leptospira kirschneri* was detected in *Arvicanthis niloticus* and *Cricetomys gambianus*	[46]
Nigeria	2012	2014	Bovine tuberculosis	Cattle	Ziehl-Neelsen test, duplex PCR	168 lung samples	Prevalence of *Mycobacterium tuberculosis* was 21.4% (AFB test) and 16.7% (duplex PCR), 81.8% of lungs with lesions were positive whiles 6.7% of lungs without lesions were positive for AFB.	[58]
Nigeria	2012-2013	2014	*Bartonella* Species	Bats and Bat Flies	qPCR, DNA sequencing	148 bats and 34 bat flies samples	51.4% of bat blood samples and 41.7% of bat flies tested were positive for *Bartonella* spp. DNA. The prevalence by culture of *Bartonella* spp. among 5 bat species ranged from 0% to 45.5%.	[67]
Nigeria	2014	2015	Bovine tuberculosis (*Mycobacterium bovis*)	Cattle	PCR, Ziehl–Neelsen (ZN) staining	800 slaughtered cattle samples	120 samples classified as suspected bTB at postmortem, 29.2% and 8.3% of which were bTB-positive by ZN and PCR respectively	[56]
Nigeria	2007–2012	2016	Bovine tuberculosis	Cattle	N/A	52, 262 slaughtered cattle samples	11.2% showed signs of tuberculosis lesion at post mortem. Average yearly prevalence of bTB was 9.1%.	[57]
Nigeria	2011, 2015	2018	*Coxiella burnetii* and *Rickettsia conorii*	Rodents, fleas	PCR	194 peridomestic rodents, and 32 associated ectoparasites	2.1% of rodents carried *C. burnetii* DNA. All ectoparasites negative for *C. burnetii* by PCR, 6.3% of the pools of various ectoparasites were positive for *Rickettsia* spp. *gltA* PCR amplification	[54]
Senegal	2008–2009	2010	*Rickettsia felis*	Humans	qPCR	204 samples from 134 patients	Prevalence of spotted fever in all samples was 4.4% (9/204)	[61]
**EASTERN AFRICA**								
Ethiopia	2007–2008	2011	Brucellosis	Cattle	RBT, CFT	1623 cattle sera	3.5% and 26.1% of animals and herds tested respectively had anti-*Brucella *antibodies.	[32]
Ethiopia	2011–2014	2015	Spotted fever group (SFG) rickettsiae	Ixodid ticks collected from domestic animals	Quantitative PCR (qPCR) system targeting the *glt*A gene	767 ixodid ticks	*Rickettsia africae* DNA was detected in 30.2% of Amblyommma variegatum, 28.6% *Am. gemma*, 0.8% *Am. cohaerens*	[59]
Ethiopia	2013	2016	Salmonellosis/*Salmonella* spp.	Dairy cattle	Culture, biochemical tests, PCR, antimicrobial susceptibility testing, serotyping and phage typing	1203 faecal samples	30 samples positive for *Salmonella*. Standard serological agglutination tests identify 9 different serotypes, with *Salmonella typhimurium* (23.3 %) being the most dominant	[73]
Ethiopia	2015	2017	Salmonellosis/*Salmonella* spp.	Dogs	Culture, antimicrobial susceptibility testing, serotyping and phage typing	360 dogs	42 (11.7%) *Salmonella*-positive. 14 serotypes detected	[74]
Kenya	2009	2010	Salmonellosis/*Salmonella* spp.	Pigs	Biochemical tests, serotyping, phage typing and PCR	116 samples	13.8% positive for *Salmonella,* 35.7% of isolates displayed antimicrobial resistance, 7.1% displayed multidrug resistance	[72]
Kenya	2012–2013	2015	Brucellosis	Humans and animals (cattle, sheep, camels, and goats)	ELISA	1088 households surveyed. 11,028 livestock (37% goats, 28% sheep, 27% cattle, and 8% camels) were sampled	Individual human and animal seroprevalence were 16 and 8% respectively. Household and herd prevalence ranged from 5–73%, and 6–68%, respectively	[38]
Kenya	2014–2015	2016	Brucellosis	Humans	Modified Rose Bengal Plate Test (RBPT), ELISA, PCR.	1067 patients	146/1067 (13.7%) tested positive for brucellosis. *B. abortus* the only *Brucella* species found using species-specific qPCR	[20]
Kenya	2014–2015	2016	Q fever	Humans	ELISA, IFA, qPCR	1067 patients	19.1% of sera were seropositive by qPCR. 16.2% of patients had acute Q fever.	[23]
Kenya	2016	2016	Q fever	Humans and cattle	ELISA	2049 human serum and 955 cattle serum samples	Overall seroprevalence of *Coxiella burnetii* was 10.5% in cattle and 2.5% in humans	[52]
Kenya	2013–2014	2017	Novel Rickettsia	Adult ticks, nymphs and larvae	PCR	4297 questing ticks	*Anaplasma phagocytophilum* detected in *Rh. maculatus* ticks and a first-time detection of *Ehrlichia chaffeensis*, *Coxiella* sp., *Rickettsia africae* and *Theileria velifera* in *Am. eburneum* ticks	[62]
Kenya	2014–2015	2017	Tularaemia (*Francisella tularensis*)	Humans	ELISA and Western blot	730 patients	71 (9.7%) were seropositive for *F. tularensis* by ELISA but 27 (3.7%) were confirmed by Western blotting	[30]
Madagascar	2010–2012	2014	*Leptospira*	Small mammals	PCR	344 samples	44 samples (12.8%) positive for *Leptospira* spp.	[44]
Madagascar	2011–2013,	2017	Brucellosis (*Brucella* spp.), Q fever (*Coxiella burnetii*) and melioidosis (*Burkholderia pseudomallei*)	Human, cattle and ticks	Specific quantitative real-time PCR assays (qPCRs)	1020 blood samples from febrile patients, 201 Zebu cattle serum samples and 330 zebu cattle-associated ticks	15 (1.5%) of samples were *Brucella*-positive, and 0% for *C. burnetii* and *Bu. Pseudomallei.* Anti-*C. burnetii* antibodies detected in 4 zebu serum samples, but no anti-*Brucella* antibodies were detected, 1% of ticks analysed tested positive for *C. burnetii* DNA.	[40]
Madagascar, Union of the Comoros	2012	2012	*Leptospira* spp.	Bats	qPCR	129 bats (52 from Madagascar and 77 from Union of the Comoros)	25 samples were positive by probe-specific qPCR. There were 34.6% and 11.7% infection rates in bats from Madagascar and Comoros, respectively.	[43]
Malawi	2011	2014	Brucellosis and bovine tuberculosis (bTB)	Cattle	Competitive ELISA, tuberculin skin test	156 and 95 cattle respectively tested for brucellosis and bTB	7.7% and 1.1% of the 156 and 95 cattle respectively tested positive for brucellosis and bTB	[42]
Mozambique	2012–2015	2017	Leptospirosis	Humans	ELISA, microagglutination test (MAT)	373 paired serum samples from febrile patients	1.3% had acute leptospirosis (MAT > 400), 10.2% had a presumptive infection (IgM-positive/MAT <400).	[26]
Tanzania	2007–2008	2011	Leptospirosis	Humans	MAT, blood culture	870 patients	8.8% of 453 paired (acute and convalescent) sera samples were confirmed leptospirosis, 3.6% of 832 patients (with ≥ 1 serum sample available) classified as having probable leptospirosis.	[27]
Tanzania	2007–2008	2011	Q Fever, Rickettsioses (Spotted Fever Group, SFGR and Typhus Group, TGR)	Humans	ELISA, culture	870 patients, 483 tested for acute Q fever, 450 tested for acuteSFGR and TGR	Infection rates of acute Q fever, SFGR and TGR were 5.0%, 8.0% and 0.5% respectively.	[25]
Tanzania	2007–2008	2012	Brucellosis	Humans	Blood culture, MAT	870 patients	455 (52.3%) had paired sera available. 16/455 (3.5%) were confirmed brucellosis, 830 people had ≥ 1 serum sample of which 0.5% had probable brucellosis	[21]
Tanzania	2013	2015	Leptospirosis, brucellosis	Humans	MAT, IgM and IgG ELISA	370 patients	11.6% had presumptive acute leptospirosis, whiles 7.0% and 15.4% showed presumptive acute brucellosis due to *B. abortus* and *B. melitensis*, respectively.	[22]
Tanzania	2011–2012	2015	*Campylobacter*	Humans	Culture, matrix-assisted laser desorption/ionisation–time-of-flight (MALDI-TOF) mass spectrometry and PCR	1195 persons	11.4% *Campylobacter*-positive. *C. jejuni* (84.6%) was most abundant *Campylobacter* species, with *C. coli* being 15.4%.	[76]
Tanzania	2012–2014	2018	Leptospirosis	Rodents, cattle, goats, sheep	qPCR, culture, phylogenetic analysis	452 cattle, 167 goats, 89 sheep	7.08% of cattle, 1.20% of goats and 1.12% of sheep carried pathogenic Leptospira infection. No pathogenic Leptospira infection was found in rodent species sampled	[47]
Uganda	2014	2014	Brucellosis	Humans	Rapid Plate Agglutination Test, Standard Tube Agglutination Test (STAT), cELISA	329 individuals (161 exposed cattle keepers and 168 individuals attending HIV testing).	Brucellosis seroprevalence in exposed cattle keepers and consumers of raw milk were 5.8% and 9%, respectively.	[36]
Uganda	2012–2013	2016	Brucellosis	Pigs	ELISA, CFT	1665 serum samples	3 samples *Brucella*-positive by ELISA, which were in turn *Brucella*-negative by CFT. SAT detected anti-*Yersinia* enterocolitica antibodies in 2 samples	[34]
Uganda	2008–2016	2017	Plague (*Yersinia pestis*)	Humans	Culture, bacteriophage lysis	255 suspected cases	78 (31%) as confirmed per specified criteria	[29]
Zambia	2011	2012	Anthrax	Humans, hippopotamuses	Culture, PCR	56 samples from human patients, hippopotamuses and soil.	30.4% of samples were culture-positive. All isolates tested were resistant to vancomycin while isolates showed 100% susceptibility to mostly the penicillins	[71]
Zambia	2008	2014	*Anaplasma phagocytophilum*, *Rickettsia* spp.	Yellow baboons (*Papio cynocephalus*) and vervet monkeys (*Chlorocebus Pygerythrus*)	PCR	88 spleen DNA samples	*Anaplasma phagocytophilum* and *Rickettsia* spp. were detected in 12 (13.6%) and 35 samples (39.8%) respectively.	[63]
Zambia	2016	2018	Anaplasmosis (*Anaplasma platys*)	Dogs	PCR	301 blood samples	9% prevalence of *Anaplasma* species	[64]
Zimbabwe	2014	2014	*Anaplasma phagocytophilum*.	Lions (*Panthera leo*), Southern African wildcats, cheetahs (*Acinonyx Jubatus*) and servals	PCR	98 whole blood samples from 86 lions, 6 Southern African wildcats, 4 cheetahs and 2 servals.	Mixed infection of *A. phagocytophilum* with other parasitic pathogens observed in 1 serval and 1 Southern African wildcat.	[66]
**SOUTHERN AFRICA**								
Botswana	2009–2012	2014	Leptospirosis (*Leptospira interrogans*)	Banded mongoose (*Mungos mungo*), Selous’ mongoose (*Paracynictis selousi*)	PCR	42 samples (41 banded mongooses and 1 Selous’ mongoose	41.5% prevalence among banded mongoose, the one Selous’ mongoose sample was Leptospira-positive	[45]
Botswana	2017	2018	*Campylobacter* spp.	Humans, chickens	Culture, whole genome sequencing	20 human samples, 70 chicken samples	Phylogenetic analysis showed a high a level of relatedness between *Campylobacter* isolated from human and various poultry sources. Resistance determinants found include *tetO* (52%), *gyrA*-T86I (47%) and *bla_OXA-61_* (72%)	[77]
South Africa	Not stated	2017	*Coxiella burnetii*, *Ehrlichia canis*, *Rickettsia* species and *Anaplasma phagocytophilum*-like bacterium	*Rhipicephalus sanguineus*, *Haemaphysalis elliptica* and *Amblyomma hebraeum*	PCR	318 ticks from dogs and cats. 147 pooled DNA samples	Prevalence were 37% (*Rickettsia* spp.), 41% (*Coxiella burnetii*), 18% (*Ehrlichia* or *Anaplasma*), 18% (*Anaplasma phagocytophilum*-like bacterium) from pooled DNA samples	[53]
**STUDIES IN DIFFERENT GEOGRAPHIC LOCATIONS**								
Senegal, Mali, Tunisia, Algeria, Gabon, and Morocco	2008, 2010–2012	2014	Q fever (*Coxiella burnetii*)	Humans	qPCR to amplify the *IS1111* and *IS30A* spacers	1888 febrile patients (1238 from Senegal, 100 from Mali, 50 from Gabon, 184 from Tunisia, 268 from Algeria and 48 from Morocco), 500 nonfebrile samples	0.3% *C. burnetii* infection rate in Algeria, 0.5% in Senegal. No infection detected in Mali, Morocco, Gabon, and Tunisia. All nonfebrile samples were negative.	[24]
